# Melanoma Cell Colony Expansion Parameters Revealed by Approximate Bayesian Computation

**DOI:** 10.1371/journal.pcbi.1004635

**Published:** 2015-12-07

**Authors:** Brenda N. Vo, Christopher C. Drovandi, Anthony N. Pettitt, Graeme J. Pettet

**Affiliations:** 1 School of Mathematical Sciences, Queensland University of Technology (QUT), Brisbane, Australia; 2 ARC Centre of Excellence for Mathematical & Statistical Frontiers (ACEMS), QUT, Brisbane, Australia; 3 Institute for Future Environments, Science and Engineering Centre, QUT, Brisbane, Australia; University of Oxford, UNITED KINGDOM

## Abstract

*In vitro* studies and mathematical models are now being widely used to study the underlying mechanisms driving the expansion of cell colonies. This can improve our understanding of cancer formation and progression. Although much progress has been made in terms of developing and analysing mathematical models, far less progress has been made in terms of understanding how to estimate model parameters using experimental *in vitro* image-based data. To address this issue, a new approximate Bayesian computation (ABC) algorithm is proposed to estimate key parameters governing the expansion of melanoma cell (MM127) colonies, including cell diffusivity, *D*, cell proliferation rate, *λ*, and cell-to-cell adhesion, *q*, in two experimental scenarios, namely with and without a chemical treatment to suppress cell proliferation. Even when little prior biological knowledge about the parameters is assumed, all parameters are precisely inferred with a small posterior coefficient of variation, approximately 2–12%. The ABC analyses reveal that the posterior distributions of *D* and *q* depend on the experimental elapsed time, whereas the posterior distribution of *λ* does not. The posterior mean values of *D* and *q* are in the ranges 226–268 µm^2^h^−1^, 311–351 µm^2^h^−1^ and 0.23–0.39, 0.32–0.61 for the experimental periods of 0–24 h and 24–48 h, respectively. Furthermore, we found that the posterior distribution of *q* also depends on the initial cell density, whereas the posterior distributions of *D* and *λ* do not. The ABC approach also enables information from the two experiments to be combined, resulting in greater precision for all estimates of *D* and *λ*.

## Author Summary

Quantifying the underlying parameters that drive the expansion of melanoma cell colonies such as the cell diffusivity, cell proliferation rate and cell-to-cell adhesion strength can improve our understanding of melanoma biology and its response to treatment. We combine a simulation-based model of collective cell spreading with a novel Bayesian computational algorithm to estimate these parameters from carefully chosen summaries of collective cell image data and to quantify their associated uncertainty across different experimental conditions. Our summarisation of the image data leads to precise estimates for all parameters. Our analysis reveals that the cell diffusivity and the cell-to-cell adhesion strength estimates depend on experimental elapsed time. Furthermore, the cell-to-cell adhesion strength estimate appears to depend on the initial cell density, whereas the cell proliferation rate estimate is approximately the same over different experimental conditions.

## Introduction

Skin cancer consists of two groups: melanoma and non-melanoma. Melanoma is the least common, approximately 5% of all skin cancer occurrences, but it is responsible for most skin cancer deaths [1]. It is estimated that 132,000 new cases of melanoma are reported worldwide each year, with more than 12,500 of these cases reported in Australia [2]. During the early stage of the disease, melanoma colonies grow and spread laterally within the epidermis. Thus, quantifying the underlying mechanisms that drive the expansion of melanoma cell colonies such as motility, proliferation, and cell-to-cell adhesion can improve our understanding of melanoma biology and its response to treatment.

Although much progress has been made in terms of developing and analysing mathematical models of expanding cell colonies, far less progress has been made in terms of understanding how to estimate model parameters including the cell diffusivity, *D*, the cell proliferation rate, *λ*, and the cell-to-cell adhesion, *q*, from experimental *in vitro* image-based data. Obtaining precise estimates of *D*, *q* and *λ* is important for developing a systematic approach to assessing the effectiveness of a potential treatment [3]. Several studies have investigated the *in vitro* expansion of cell colonies using partial differential equations [4–7]. These approaches are limited in that they provide point estimates, and the uncertainty in the estimate is not quantified. An alternative modelling approach uses discrete, individual-based models [8–10], which can incorporate several important biological factors such as cell heterogeneity [11]. Discrete models can also produce discrete image-based and video-based information which is ideally suited to collaborative investigations involving applied mathematicians and experimental cell biologists. However, the likelihood functions for these discrete models are generally intractable, so standard statistical inferential methods for these models are not applicable.

To overcome these issues, an approximate Bayesian computation (ABC) approach is developed to jointly infer the values of *D*, *q* and *λ* from a discrete stochastic model describing the expansion of cell colonies. ABC is a well established method that has been successfully applied in a wide range of areas such as population genetics [12], infectious diseases [13, 14], astronomical model analysis [15] and cell biology [16]. Generally, ABC approximates the likelihood function by model simulations, the outcomes of which are compared with the observed data [16, 17]. In this paper, we propose a new ABC algorithm that is shown to be more efficient than state-of-the-art algorithms available in the literature [17–20] by developing a new sequential Monte Carlo approach. ABC requires the specification of a set of summary statistics to compare the observed and simulated data. Each of our experimental datasets is initially summarised using a high dimensional vector of summary statistics (hereafter referred to as the pilot summary statistics). Unfortunately, ABC is not able to handle high dimensional summary statistics in an efficient manner [21], so we adopt a semi-automatic approach [22] to reduce the dimension of the pilot summary statistics. Using a synthetically generated dataset, we demonstrate that combining our new ABC algorithm and the derived set of summary statistics can precisely recover all parameters.

We apply this procedure to the experimental data of human malignant melanoma cells (MM127) in a barrier assay [23] in two different experimental scenarios: (1) Mitomycin-C is applied as a treatment to suppress cell proliferation, and (2) no treatment is applied. We aim to obtain a joint approximate posterior distribution for *D*, *q* and *λ* for different combinations of initial cell densities, *C*(0), and experimental times, *T*, in each scenario. Through the ABC analyses, the associated uncertainty in the parameter values is quantified and interpreted in terms of the coefficient of variation (CV) and probability intervals of the posterior distribution. Thus, our work adds significant extra information about model parameters relative to the previous analysis [23], which obtained point estimates of *D*, *q* and *λ* separately. In the previous analysis [23], *D* and *q* were estimated only from the experiments with cell proliferation suppressed.

Previous approaches often assume that these parameter values are the same over different experimental conditions [3, 23, 24]. The findings from this study show that the posterior estimate of *D* appears to depend on experimental time and weakly depend on the initial cell density, which is consistent with the results reported in Vo et al. [16] for 3T3 fibroblast cells. A similar trend of dependency is also found for *q*; but in contrast the posterior estimates of *λ* remain similar over time. These results suggest that a more complicated model might be warranted. However, this finding could not have been achieved without first exploring the suitability of the standard model under consideration here.

The experimental data analysed in Vo et al. [16] also consists of two separate scenarios, with and without Mitomycin-C pre-treatment. Vo et al. [16] demonstrate that *λ* cannot be identified by leading edge data solely, unless prior information about *D* (obtained from the experiment with the treatment applied) is incorporated via a sequential Bayesian learning approach. In this paper, we show that all parameters (including *λ*) can be estimated precisely through the inclusion of additional summary statistics (cell densities and percentages of isolated cells) even when only vague prior information is specified for parameter values. Nonetheless, we show that the Bayesian sequential learning approach [16] is still useful here as we are able to obtain greater precision of the parameter values.

## Materials and Methods

### Experimental data and image analysis

The details of the experimental method were described previously [23]. Briefly, monolayers of human malignant melanoma cells (MM127, [25, 26]) were cultured in 24-well tissue culture plates, where each well had a diameter of 15.6 mm. Experiments were conducted in two different experimental scenarios: (1) with Mitomycin-C pre-treatment to suppress cell proliferation, and (2) without Mitomycin-C pre-treatment. Mitomycin-C, an alkylating antibiotic, is used to block DNA and RNA replication and protein synthesis. Thus, given an appropriate concentration, Mitomycin-C inhibits mitosis and proliferation of several cell types [27]. For the melanoma experiments here, 10 µg ml^−1^ Mitomycin-C was added to the cells one hour prior to transfer to the wells [23].

To initiate each experiment, either 20,000 or 30,000 cells were approximately evenly distributed within a circular barrier, of diameter 6.0 mm, located at the centre of the well. After allowing the cells to attach for 1 h, the barriers were lifted and population-scale images were recorded at either 24 h or 48 h, independently. To extract detailed information about the location of individual cells in the population, high magnification images of a transect across the centre of the cell population were also acquired, where the nuclei were stained with Propidium Iodide (PI). Furthermore, each experimental scenario, for each initial cell density and each termination time, was repeated three times. Thus, a 2 × 2 × 2 balanced experimental design was conducted with three replicates, producing a total of 24 independent experimental images of expanding cell colonies and the corresponding transect images.

**Fig 1:**
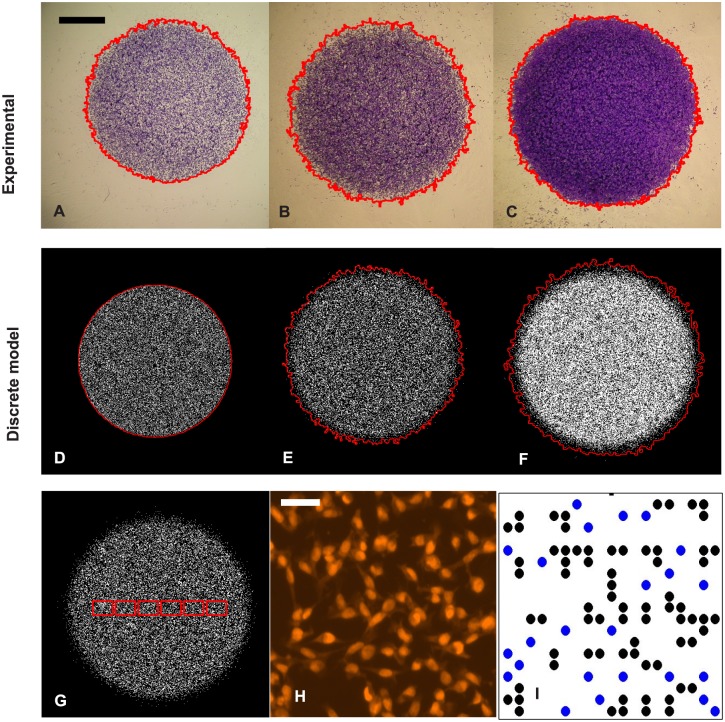
Experimental data, discrete model simulations and image analysis. Subfigures A-C correspond to experimental images of entire melanoma cell colonies for 30,000 cell experiments at time 0 h, 48 h in Scenario 1 (with Mitomycin-C pre-treatment) and 48 h in Scenario 2, respectively, with the detected leading edges superimposed (red curve). The scale bar in A represents 2 mm. Subfigures D–F are the snapshots of the discrete models with the same experimental condition as A–C. Subfigure G shows the positions of six sub-regions (red rectangles) in a simulated experiment, each red rectangle contains 39 × 28 lattice sites. An experimental snapshot which shows positions of individual cells, is presented in subfigure H. This image is from a 30,000 cell experiment, terminated at 48 h, in Scenario 1. The scale bar in H corresponds to 50 µm. The cell positions are then mapped to a square lattice in subfigure I with blue dots identifying the isolated cells.

From preliminary analysis, we note that cell colonies maintain an approximately circular shape during the experiments. Thus, for each population-scale image, which shows the spatial expansion of the entire melanoma cell colony, we detect the position of the leading edge, then estimate the radius of the colony by converting the area enclosed by the leading edge to the equivalent circular radius, *R*, using a segmentation algorithm written with the Matlab Image Processing Toolbox [16, 28] (Table S1 in S1 Text). We use the exact same edge detection algorithm for both our experimental data and the images produced by the discrete simulation model described in the next section. Images in Fig 1A–1C show the entire expanding cell colonies for the 30,000 initial cell experiments at time 0 h and 48 h where cells were pre-treated with Mitomycin-C, and 48 h without the treatment, respectively, together with the estimated leading edge superimposed.

To extract cell densities and measure cell clustering, we mapped the position of the cells to a square lattice with spacing Δ = 18 µm (Fig 1H and 1I), which corresponds to an average diameter of the cell nucleus [23]. For each experiment, we analyse six sub-regions along a transect image (Fig 1G). Each sub-region has size 700 × 500 µm or 39 × 28 lattice sites. We then count the number of cells in each sub-region, ${\left\{c_{i}\right\}}_{i=1}^{6}$, together with the proportion of isolated cells, ${\left\{p_{i}\right\}}_{i=1}^{6}$. A cell is identified as isolated if all of its nearest neighbours (north, south, east and west) are unoccupied. For each experiment at each initial cell density and termination time, at each sub-region, we average *c*_*i*_ and *p*_*i*_ over three replicates (Tables S2 and S3 in S1 Text).

**Fig 2:**
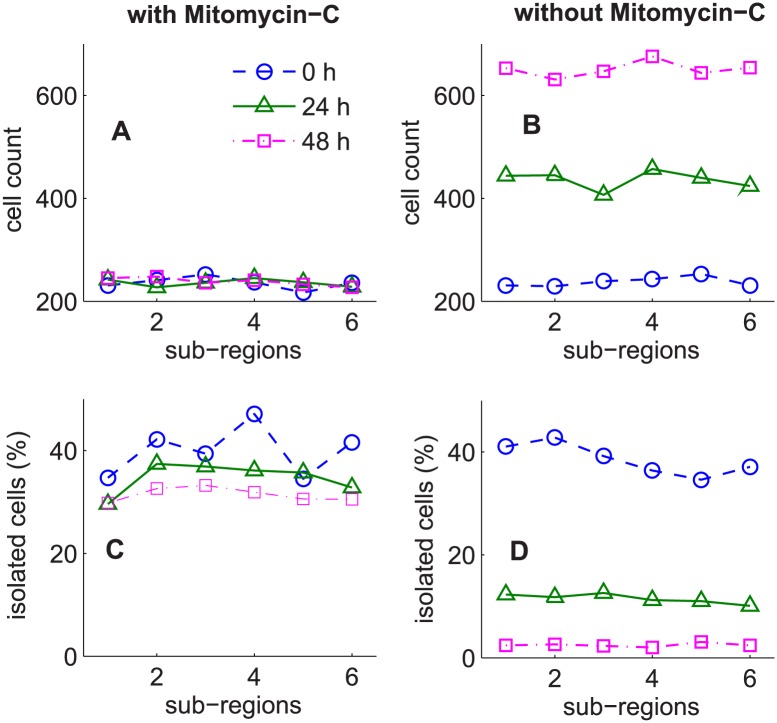
Cell counts and percentages of isolated cells. Results correspond to experiments initiated with 20,000 cells. Subfigures A–B, C–D show the cell counts and the percentages of isolated cells for the six sub-regions after averaging over the three replicates, for the experiments with and without Mitomycin-C pre-treatment, respectively. The dashed line (circle markers), the solid line (triangle markers) and the dashed dotted line (square markers) correspond to the experiment at initial time, 0 h, terminated at 24 h and 48 h, respectively.

Summaries of ${\left\{c_{i}\right\}}_{i=1}^{6}$ and ${\left\{p_{i}\right\}}_{i=1}^{6}$ (average over the three replicates) for experiments initialised with 20,000 cells are given in Fig 2. We observe that, for experiments where cells were not pre-treated with Mitomycin-C (Fig 2B), ${\left\{c_{i}\right\}}_{i=1}^{6}$ increases significantly over time, whereas the differences in ${\left\{c_{i}\right\}}_{i=1}^{6}$ for the corresponding experiments (Fig 2A), where cell proliferation was suppressed, are minimal. Furthermore, ${\left\{p_{i}\right\}}_{i=1}^{6}$ (Fig 2C and 2D) appear to decrease over time which suggests that melanoma cells possibly form more clusters as the experiments proceed. These trends are consistent with previous research [23], which shows that cell-to-cell adhesion plays an important role in the melanoma expanding colonies.

### Discrete stochastic model

To describe the expansion of a single layer of melanoma cell colonies, we employ a discrete lattice based model that incorporates cell migration (unbiased random walk), cell proliferation and cell-to-cell adhesion. The discrete model here is similar to the model used in [9, 16, 23]. We incorporate a volume exclusion process and realistic crowding effects [8, 9, 29], so each lattice site can be occupied by at most one agent.

To simulate the experiments, we use a two-dimensional square lattice of size 867 × 867, with lattice spacing Δ = 18 µm, so that the width of the lattice corresponds to the diameter of the well, 15.6 mm (15600 µm/18 µm = 867). Let *C*(*t*) be the number of agents in the discrete model at time *t*, *P*_*m*_ ∈ [0, 1] be the probability that an isolated agent will attempt to step a distance Δ within a time step of duration *τ*, and *P*_*p*_ ∈ [0, 1] represent the probability that an agent will attempt to proliferate and deposit a daughter within a time step of duration *τ*. The strength of cell-to-cell adhesion is represented by *q* ∈ [0, 1].

Initially, *C*(0) agents (20,000 or 30,000 agents) are placed randomly inside a circle which has a radius of 177 lattice sites, corresponding to the mean radius of the experimental observations at time *t* = 0 h. We use an approximate random sequential update (RSU) algorithm [30, 31] to perform the simulations. To step from time *t* to time *t* + *τ*, *C*(*t*) agents are sampled, with replacement, and given the opportunity to move with probability *P*_*m*_ × (1 − *q*)^*n*^, where 0 ≤ *n* ≤ 4 is the number of occupied nearest neighbour sites. If an agent is at position (*x*, *y*) and has an opportunity to move, it will attempt to step to either (*x* ± Δ, *y*) or (*x*, *y* ± Δ), with each target site chosen with equal probability. The higher the value of *q*, the more difficult it is for an agent to move away from its neighbours.

A similar mechanism is employed for proliferation events. A proliferative agent at position (*x*, *y*) will attempt to deposit a daughter agent at (*x* ± Δ, *y*) or (*x*, *y* ± Δ), with each target site chosen with equal probability. Since the model is an exclusion process, any attempted motility or proliferation event that would place an agent on an occupied site is aborted (S1 Text, Algorithm S1). We do not consider any death mechanism in this model since there was no evidence of any cell death in the experiment [23]. Given the termination time, *T* (24 h or 48 h), the model requires *T*/*τ* time steps.

The cell expanding colonies are governed by three parameters (*P*_*m*_, *q*, *P*_*p*_). These parameters are related to the cell diffusivity, *D*, and the proliferation rate, *λ*, by *D* = *P*_*m*_ Δ^2^/4*τ* and *λ* = *P*_*p*_/*τ*, respectively [29], with Δ and *τ* set fixed. In this work, we apply our new ABC algorithm to obtain joint posterior distributions for (*P*_*m*_, *q*, *P*_*p*_), then use these relationships and the values of Δ and *τ*, to rescale posterior distributions of *P*_*m*_ and *P*_*p*_ into posterior distributions of *D* and *λ*, respectively.

We note that the RSU algorithm is an approximation of the exact, continuous time Gillespie algorithm [32]. The value of the time duration *τ* is a trade-off between the accuracy of the approximation and the computational time to simulate the experiments. To choose a suitable value for *τ*, we perform 100 model simulations using the same diffusion coefficient *D* = 220 µm^2^h^−1^, obtained with different pairs of parameters (*τ* = 0.1 h, *P*_*m*_ = 0.2716), (*τ* = 0.08 h, *P*_*m*_ = 0.2173), (*τ* = 0.06 h, *P*_*m*_ = 0.1630), (*τ* = 0.04h, *P*_*m*_ = 0.1086) and (*τ* = 0.02 h, *P*_*m*_ = 0.0543). We then compare the plots of the probability density of the resulting radii, percentages of isolated cells and total number of cells in six sub-regions. We found that there is a negligible difference between results from simulations with *τ* = 0.04 h and *τ* = 0.02 h. This means that *τ* = 0.04 h is small enough to produce reasonably accurate simulations. Therefore, for all model simulations hereafter, we use *τ* = 0.04 h. Snapshots of the discrete stochastic models initialised with 30,000 agents and termination time at 0 h, 48 h in Scenario 1, and 48 h in Scenario 2 are shown in Fig 1D–1F, respectively.

In this paper, we do not have any measurement for the uncertainty in *C*(0). Thus, all of the simulations from the discrete models use the same initial values of *C*(0), i.e. 20,000 cells or 30,000 cells. However, if we have this measurement, we can easily incorporate it in the ABC algorithms by drawing the value of *C*(0) from its distribution before proceeding to simulate a realisation of the model.

### Approximate Bayesian computation

The discrete models described above can incorporate realistic cell behaviour. However, their likelihood functions are not available in an analytical form and are not computationally tractable, so standard statistical inferential methods for these models are not applicable. Combining ABC and the discrete stochastic model is a promising approach since ABC bypasses the evaluation of the likelihood by a simulation-based procedure [12, 17]. The aim of the ABC approach is to find the joint approximate posterior distributions, which are the distributions of the unknown parameters given the observed summarisation of the data and the prior information. All inferences about the parameters including point estimates and probability intervals are made from the posterior distributions.

Let *y*_*obs*_ and *y*_*sim*_ represent the observed and the simulated data, *θ* = (*P*_*m*_, *q*, *P*_*p*_) represent the vector of unknown parameters and *π*(*θ*) be the prior distribution for *θ*. We define a distance metric *ρ* which is a function of *y*_*obs*_ and *y*_*sim*_, *ρ* = *ρ*(*y*_*obs*_, *y*_*sim*_). ABC approaches consist of four major steps: sampling a proposed parameter *θ* ^⋆^, simulating data as per the observed data structure from the model with *θ* ^⋆^, comparing *y*_*sim*_ with *y*_*obs*_ by computing *ρ* = *ρ*(*y*_*obs*_, *y*_*sim*_) and accepting the proposed *θ* ^⋆^ if *ρ*(*y*_*obs*_, *y*_*sim*_) ≤ *ϵ*, where *ϵ* ≥ 0 is a tolerance value. The accepted sample of parameter values forms the approximation of the posterior distribution of the model parameters. The choice of *ϵ* is a trade-off between accuracy and computational effort. In practice, different ABC algorithms have different approaches to sample the values of *θ* ^⋆^.

ABC rejection is the simplest ABC algorithm, which generally samples *θ* ^⋆^ from the prior distribution. This algorithm is easy to implement and is embarrassingly parallel. However, for complex models where the prior distribution is substantially different from the posterior, this approach results in low acceptance rates and is computationally inefficient. Vo et al. [16] employed the ABC rejection algorithm to estimate *D* and *λ*, using the leading edge data of 3T3 fibroblast cell populations. This study samples a large number of proposed parameters from the prior, each with a corresponding artificial dataset and a value of discrepancy *ρ*. These parameters are then sorted by their discrepancies and only a small proportion of parameters with the lowest discrepancy are retained. In the study of Vo et al. [16], a uniform prior was used, suggesting that for a reasonably low *ϵ*, the proportion of parameters being kept is very small, approximately 0.1%. Thus, this study suggests that it is necessary to generate 10^6^ model simulations to obtain an ABC posterior sample of size 1,000.

Several studies [33–35] proposed a Markov chain Monte Carlo approach to ABC (MCMC-ABC). MCMC-ABC algorithms make local proposals in high (ABC) posterior support regions, thus they can improve the acceptance rates. However, the posterior samples are highly correlated and the algorithms can easily be trapped in regions of low posterior density [35]. Another class of ABC is SMC-ABC which was pioneered by [36] to overcome the problems associated with ABC rejection and MCMC-ABC. SMC-ABC algorithms involve sampling from a sequence of ABC posterior distributions with a non-increasing sequence of tolerances, ${\left\{\epsilon_{k}\right\}}_{k=1}^{M}$. Thus, this last class of ABC only draws proposed parameters in sequentially higher posterior support regions, rather than the entire parameter space. A review of ABC algorithms can be found in [37].

In this paper, we only focus on SMC-ABC algorithms. Instead of drawing a proposed value *θ* ^⋆^ one at a time, the SMC algorithms work with a large set of parameter values simultaneously and treat each parameter vector as a particle. The particles are moved and filtered at each stage of the algorithm. Initially, a set of *N* particles, ${\left\{\theta_{i}\right\}}_{i=1}^{N}$, is often sampled from the prior distribution *π*(*θ*) and each sampled particle has an equal weight of 1/*N*. To propagate a particle from iteration *k* − 1 to iteration *k*, SMC-ABC algorithms involve three steps: (i) re-sampling: a sampled particle candidate *θ* ^⋆^ is chosen randomly from the set of particles at *k* − 1 with probability proportional to their weights, $\theta^{⋆}∼{\left\{\theta_{i}^{k-1},W_{i}^{k-1}\right\}}_{i=1}^{N}$; (ii) perturbing: the particle candidate *θ* ^⋆^ is perturbed by a transition kernel to propose a new particle *θ* ^⋆⋆^, *θ* ^⋆⋆^ ∼ *K* ^*k*^(⋅|*θ* ^⋆^), and (iii) simulating *y*_*sim*_ from the model, *y*_*sim*_ ∼ *f*(⋅|*θ* ^⋆⋆^). To maintain *N* particles throughout the algorithm, the steps (i-iii) are repeated until a parameter value is found such that the condition *ρ*(*y*_*obs*_, *y*_*sim*_) ≤ *ϵ*_*k*_ is satisfied. Different SMC algorithms can be distinguished by the transition kernel, the schedule of the tolerances and how sampling weights are assigned to the particles.

In the literature, there are several versions of SMC-ABC algorithms. For example, SMC-ABC algorithms of [18, 19, 38] use a Gaussian Markov kernel with a covariance matrix as twice the empirical covariance matrix of the current set of particles. These algorithms also assign to each particle *θ* ^*k*^ a weight given by:

$$\begin{array}{c} W^{k}\propto \frac{\pi (\theta^{k})}{\sum_{j=1}^{N}W_{j}^{k-1}K^{k}\left(\theta^{k}|\theta_{j}^{k-1}\right)}. \end{array}$$ (1)

These algorithms have the advantage that they require fewer model simulations, although the sequence of tolerances in these algorithms is determined manually. Drovandi et al. [17] and Del Moral et al. [39] proposed an adaptive SMC algorithm that can determine a decreasing set of tolerances dynamically. This can be achieved by sorting the particles by their discrepancies and then dropping a proportion of the particles with the highest discrepancy. However, these algorithms use an MCMC kernel which has a drawback of replications of particles. To reduce this problem, Drovandi et al. [17] suggest to repeat the MCMC step (steps (ii) and (iii) above) a number of times, which also can lead to a large number of unused model simulations.

We take the advantage of fewer model simulations from SMC-ABC algorithms [18, 19, 36] and the advantage of automatically determining tolerance values from [17] (also named the SMC replenishment (RSMC) algorithm) and incorporate these in one algorithm, hereafter referred to as ASMC (S1 Text, Algorithm S2). Our ASMC algorithm is similar to that proposed in [20] (also named adaptive population Monte Carlo (APMC) algorithm) who also determine the sequence of tolerances adaptively and use the re-weighting scheme above. However, in each iteration, the APMC algorithm [20] only performs steps (i-iii) above once and keeps all the *N* particles, so the particle’s discrepancy value is not enforced to be below a particular tolerance.

In the APMC algorithm, the sequence of tolerances fluctuate, whereas the sequence of tolerances in the RSMC and ASMC algorithms is always non-increasing. Therefore, we cannot use a single indicator to compare the performance of the three algorithms. We suggest comparing the RSMC and ASMC using the final tolerance, and comparing the ASMC and APMC using the same computational effort. Using synthetically generated data, we show that our algorithm requires fewer model simulations than the RSMC algorithm [17], given the same target tolerance *ϵ*_*final*_. In addition, given the same number of simulations, our algorithm is shown to produce a lower tolerance value (thus higher accuracy) relative to the APMC algorithm [20].

#### Summary statistics

In the ABC framework, direct comparison between the observed and the simulated datasets is often inefficient, especially when the data is high dimensional [21]. Thus, several authors have considered comparing a summary statistic of the data, *S*(*y*), which has smaller dimension than the full data. The choice of summary statistics is a crucial step in the ABC approach since it involves a trade-off between information loss and dimension reduction [40].

For the application, it is impossible to use the information of the entire assay. Thus for each assay, we only estimate the leading edge and analyse six sub-regions along the transect. Our pilot summary statistic of the data $L=\{R_{\left(1\right)},R_{\left(2\right)},R_{\left(3\right)},{\left\{c_{i}\right\}}_{i=1}^{6},{\left\{p_{i}\right\}}_{i=1}^{6}\}$, where *R*_(1)_ < *R*_(2)_ < *R*_(3)_ are the ordered radii of the expanding cell colonies for three experimental replicates, is too high dimensional. This leads to a computational challenge in matching the observed and simulated summary statistics. This problem is also referred to as the curse dimensionality [21], and so the dimension of the summary statistics should be kept as small as possible to improve computational efficiency. Therefore, we employ the dimension reduction procedure [22] to generate one summary statistic (estimate of the posterior mean) per parameter. The posterior means of the parameters are estimated via regression.

The procedure is as follows: (i) perform a pilot run of ABC, using the pilot summary statistics, to find the regions of non-negligible posterior density, (ii) draw *M* samples of ${\left\{\theta_{i}\right\}}_{i=1}^{M}$ from the parameter space resulting from the pilot run, each with a corresponding artificial dataset ${\left\{y_{i}\right\}}_{i=1}^{M}$ and a summary statistic ${\left\{L_{i}\right\}}_{i=1}^{M}$, and (iii) fit a multiple linear regression model (Eq (2)) to each component of *θ* in turn:

$$\begin{array}{c} \theta_{i,j}=\alpha_{j}+\beta_{j}^{T}g_{j}\left(L_{i}\right)+\xi_{i,j},\;i=1,\cdots ,M,j=1,\cdots ,J, \end{array}$$ (2)

where *J* is the number of parameters. In the regression model, the error terms, *ξ*_*i*,*j*_, have mean zero and *g*_*j*_(⋅) is a vector-valued function, so that *g*_*j*_(*L*) is a vector of transformations of the pilot summary statistics. For the application in our model, we choose *g*_*j*_(*L*) = (*L*, *L* ^2^) and *M* = 5,000. However, for other applications, other choices of *g*_*j*_(⋅) could be considered to obtain a better fit in the regression. Here, *α*_*j*_ is the intercept parameter and *β*_*j*_ is the vector of regression coefficients. The expected value of *θ*_*j*_ given the simulated summary statistic *L*_*i*_, *E*[*θ*_*j*_|*L*_*i*_], is then estimated by $\hat{\alpha_{j}}+{\hat{\beta_{j}}}^{T}g_{j}\left(L_{i}\right)$. To find the best regression model, we employ a stepwise (bidirectional) regression method and the Bayesian information criterion (BIC) for model selection. The derived summary statistic for each parameter is then defined by $S_{j}\left(y\right)={\hat{\beta_{j}}}^{T}g_{j}\left(L\right)$, *j* = 1, …, *J*, where $\hat{\beta_{j}}$ is the estimated coefficients from the best regression model. Thus, there is only one summary statistic per parameter.

#### Discrepancy function

In an attempt to accommodate summary statistics with different scales and correlations between summary statistics, we consider the Mahalanobis distance to compare *S*_*obs*_ and *S*_*sim*_, where $S_{obs}\;=\;{\left\{S_{j}\left(y_{obs}\right)\right\}}_{j=1}^{J}$ and $S_{sim}\;=\;{\left\{S_{j}\left(y_{sim}\right)\right\}}_{j=1}^{J}$. This discrepancy function is given by

$$\begin{array}{c} \rho \left(y_{obs},y_{sim}\right)={\left(S_{obs}-S_{sim}\right)}^{T}\times W^{-1}\times \left(S_{obs}-S_{sim}\right), \end{array}$$ (3)

where *W* is an estimate of the covariance matrix of the summary statistics ${\left\{S_{j}\right\}}_{j=1}^{J}$. To estimate *W*, we simulate 100 simulated datasets ${\left\{y_{i}|{\left\{\hat{\theta_{j}}\right\}}_{j=1}^{J}\right\}}_{i=1}^{100}$, using the estimated posterior mean $\hat{\theta_{j}}\;=\;\hat{\alpha_{j}}\;+\;{\hat{\beta_{j}}}^{T}g_{j}\left(L\right)$, *j* = 1, …, *J*, obtained from the regression step above. For each simulated dataset *y*_*i*_, we compute the pilot summary statistics, then obtain the derived summary statistics ${\left\{S_{i,j}\right\}}_{j=1}^{J}$, *i* = 1, …, 100. *W* is subsequently estimated by *cov*{*S*_*i*,*j*_}, *i* = 1, …, 100 and *j* = 1, …, *J*.

## Results

### Validation and comparing algorithms’ performance

To examine the utility of our new ABC algorithm and to investigate whether the derived set of summary statistics is informative for parameter inferences, we simulated a dataset with biologically relevant parameter values (*P*_*m*_ = 0.1, *q* = 0.2, *P*_*p*_ = 0.0012), which corresponds to (*D* = 202.5 µm^2^h^−1^, *q* = 0.2, *λ* = 0.03h^−1^). The synthetic dataset has *C*(0) = 20,000 cells, *T* = 24 h and is replicated three times. This dataset represents experiments in Scenario 2.

**Fig 3:**
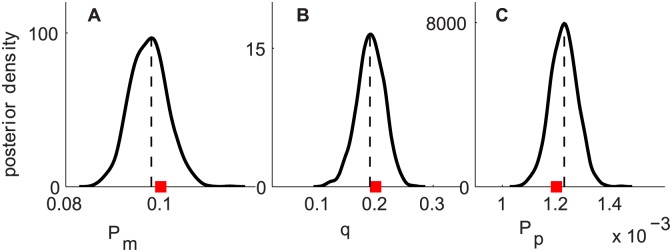
Results for synthetic data. Subfigures A–C correspond to the ABC posterior distributions of *P*_*m*_, *q* and *P*_*p*_, respectively. Uniform priors are placed on all parameters, *P*_*m*_ ∼ *U*(0,1), *q* ∼ *U*(0,1) and *P*_*p*_ ∼ *U*(0,1). The posterior means are plotted as black vertical dashed lines and the true parameter values are shown as red squares.

We first summarise the synthetic dataset in terms of the pilot summary statistics, including three radii of the expanding cell colonies for three replicates (order statistics), the numbers of cells and the percentages of isolated cells in six sub-regions along a transect after averaging over three replicates. The ABC posterior distributions resulting from the pilot run with the pilot summary statistics have significant spread. So, a multiple linear regression procedure is performed to generate one summary statistic for each parameter. We then apply the new ABC algorithm with the derived set of summary statistics and uniform priors for all parameters, *P*_*m*_ ∼ *U*(0,1), *q* ∼ *U*(0,1) and *P*_*p*_ ∼ *U*(0,1). The resulting posterior distributions for (*P*_*m*_, *q*, *P*_*p*_) are presented in Fig 3. These results show well-defined posterior distributions with narrow spread and posterior means close to the true values. The posterior correlation coefficients of (*P*_*m*_, *q*), (*q*, *P*_*p*_) and (*P*_*m*_, *P*_*p*_) are between −0.2 to 0.3. Thus, it is evident that our new ABC algorithm combined with our method for determining summary statistics allows us to recover all parameters rather precisely.

**Fig 4:**
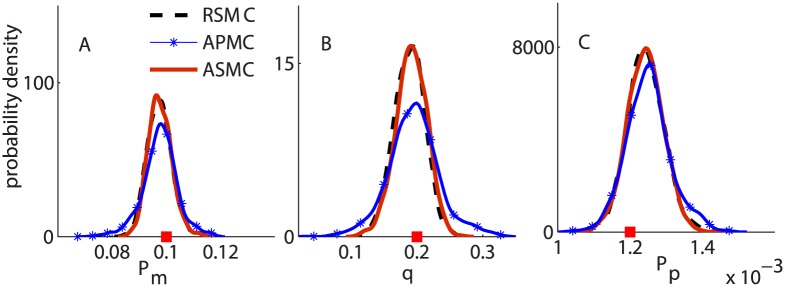
Comparing the performance of the three SMC-ABC algorithms. Subfigures A, B and C correspond to the ABC posterior distributions of *P*_*m*_, *q* and *P*_*p*_, respectively. In all subfigures, the (dashed) black, the (solid, with markers) blue and the (solid) red curves correspond to the RSMC, the APMC and the ASMC algorithms, respectively.

**Fig 5:**
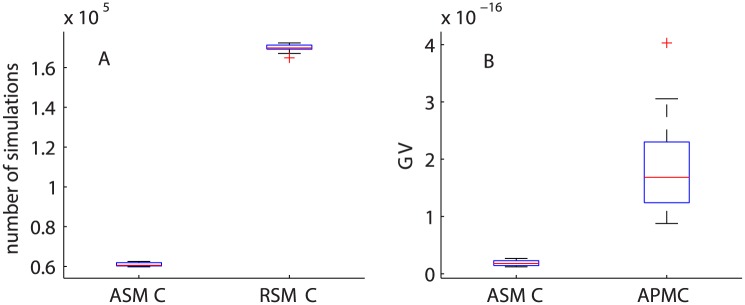
Comparing the number of model simulations and GV. (A) Boxplot of the number of model simulations for RSMC and ASMC, given the same *ϵ*_*final*_. (B) Boxplot of the GV for the resulting ABC posterior distributions from ASMC and APMC based on a similar number of model simulations.

Using the synthetically generated data, we also compare the performance of the three algorithms: RSMC, APMC and ASMC. For all algorithms, we set *N* = 1000 particles and run each algorithm 10 times to compare the resulting posterior distributions, the total number of model simulations and the generalized variance (GV, or the determinant of the posterior variance-covariance matrix). A comparison of ABC posterior distributions from the three algorithms is shown in Fig 4. For RSMC and ASMC, we set *ϵ*_*final*_ = 0.1. For all cases, the posterior distributions from RSMC (the dashed black curves) and ASMC (the solid red curves) are almost indistinguishable, however, the RSMC requires approximately 2.5 times more model simulations than the ASMC algorithm (Fig 5A).

For the APMC algorithm, we use 62 iterations (giving the total number of model simulations similar to the number of model simulations for ASMC, 62,000). Results in Fig 4 suggest that the posterior distributions from the ASMC algorithm has smaller variance than the results from the APMC algorithm (the blue curves with markers) due to the ability of ASMC in getting to a smaller value of *ϵ* with a similar computational effort. We then compute the GV of the resulting ABC joint posterior distributions from the ASMC and APMC algorithms from the 10 runs (Fig 5B). We observed that the GVs for the resulting posterior distributions from APMC are approximately three times larger than the corresponding GV from the ASMC algorithm. Thus, for this application, our algorithm performs better than the RSMC and the APMC algorithms. We now apply the ASMC algorithm to the experimental data in the two scenarios and interpret the results in terms of the biologically relevant parameters *D*, *q* and *λ*.

### Scenario 1: Experiments with Mitomycin-C pre-treatment

This section presents the results for *D* and *q* for all experimental conditions in Scenario 1, where cells were pre-treated with Mitomycin-C to suppress cell proliferation. Uniform priors are placed on all parameters, *P*_*m*_ ∼ *U*(0,1) and *q* ∼ *U*(0,1). From the regression procedure to generate one summary statistic *S* for each parameter, for all cases, we observe that all pilot summary statistics (*R*_(1)_, *R*_(2)_, *R*_(3)_, ${\left\{c_{i}\right\}}_{i=1}^{6}$ and ${\left\{p_{i}\right\}}_{i=1}^{6}$) are informative about *D*. However, to obtain estimates for *q*, only *R*_(1)_, ${\left\{c_{i}\right\}}_{i=1}^{6}$ and ${\left\{p_{i}\right\}}_{i=1}^{6}$ were significant in the regression.

The ABC estimate of the posterior expected value of *D* and *q*, *E*[*D*] and *E*[*q*], 90% credible intervals, CI, the coefficient of variation, CV, and the correlation coefficient, *r*, from all experimental conditions, are given in Table 1. To assess the accuracy of our resulting estimates from the true ABC posteriors, we computed the Monte Carlo standard error, MCSE, for *E*[*D*] and *E*[*q*] in all experimental conditions, MCSE = $\sigma /\sqrt{\text{ESS}}$ [41]. Here, *σ* is the posterior standard deviation and ESS is the effective sample size. We use Kish’s approximation method [42] to compute the ESS, ESS = $1/\sum_{i=1}^{N}W_{i}^{2}$, where *W*_*i*_ is the normalised weight for the *i* ^*th*^ parameter value. For all cases, the ABC posterior consists of 1,000 parameter values, which leads to an ESS usually in the range 700–850. Our posterior sample size leads to a small MCSE for both *E*[*D*] and *E*[*q*], less than 0.2% and 0.4% of the estimate of their expected values, respectively.

**Table 1:** ABC posterior summary for *D* and *q* for all experiments in Scenario 1. Results shown include the posterior mean (and the 90% CI in the parentheses), the coefficient of variation, CV, and the correlation coefficient, *r*.

*C*(0)	*T*(*h*)	*E*[*D*] (90%CI) µm^2^h^−1^	CV(*D*)(%)	*E*[*q*] (90%CI)	CV(*q*)(%)	*r*
20 000	0—24	225.9 (212.1, 240.3)	4.0	0.23 (0.19, 0.27)	9.9	0.4
	0—48	288.3 (273.7, 304.9)	3.3	0.29 (0.25, 0.32)	6.7	0.4
	24—48	335.9 (309.9, 366.9)	5.6	0.32 (0.28, 0.35)	6.3	0.2
30 000	0—24	251.8 (235.9, 269.6)	4.1	0.36 (0.33, 0.39)	5.4	0.5
	0—48	297.0 (279.1, 316.4)	4.1	0.47 (0.45, 0.50)	3.0	0.6
	24—48	317.7 (293.8, 344.9)	4.9	0.50 (0.47, 0.52)	3.2	0.4

From Table 1, we observe that the CV for *D* and *q* are also quite small, approximately 6% and 10%, respectively, which means that we can obtain reasonably precise estimates for *D* and *q* using the derived summary statistics. The correlation coefficient between *D* and *q* for all combinations is between 0.2 to 0.6. This suggests that multiple combinations of values of *D* and *q* can generate similar expanding cell colonies in terms of our pilot summary statistics.

For both initial cell densities (20,000 and 30,000 cells), we observe that the values of *E*[*D*] for the experiments terminated after 48 h are higher than those values for experiments terminated after 24 h. This finding suggests that estimates of *D* appear to depend on the experimental time, *T*, which is consistent with the results reported in [16] for 3T3 fibroblast cells. It is conjectured that some amount of time could be required for the cells to adjust to their new or modified environments encountered as part of the experimental protocol. The cell motility, therefore, could be reduced during this transition phase. A similar trend of dependency is also found for *q*. This motivates us to investigate the values of *D* and *q* for the period 24–48 h.

Let {*D*_(0–24)_, *q*_(0–24)_}, {*D*_(24–48)_, *q*_(24–48)_} and {*D*_(0–48)_, *q*_(0–48)_} represent the cell motility coefficient and strength of cell-to-cell adhesion for the period 0–24 h, 24–48 h and 0–48 h, respectively. Estimates of posterior distributions for {*D*_(0–24)_, *q*_(0–24)_} and {*D*_(0–48)_, *q*_(0–48)_} have already been obtained from experimental data at 24 h and 48 h, respectively. To obtain estimates for {*D*_(24–48)_, *q*_(24–48)_}, two stages of simulations are required, from 0–24 h and from 24–48 h. In the first stage, model simulations use parameter sets that are drawn from the distributions of {*D*_(0–24)_, *q*_(0–24)_}; whereas, in the second stage, the model simulations update the cell colonies with parameter sets that are drawn from the distributions of {*D*_(24–48)_, *q*_(24–48)_}. We consider two approaches to infer the values of {*D*_(24–48)_, *q*_(24–48)_}.

- Approach 1: We jointly infer the values of {*D*_(0–24)_, *q*_(0–24)_} and {*D*_(24–48)_, *q*_(24−48)_} by simultaneously comparing experimental data that are terminated at 24 h and 48 h with the simulated data at the corresponding terminated times. In this approach, we place a uniform prior on both parameter sets {*D*_(0–24)_, *q*_(0–24)_} and {*D*_(24–48)_, *q*_(24–48)_}. We observe that the ABC posterior distributions of {*D*_(0–24)_, *q*_(0–24)_} in this approach are indistinguishable with the estimates previously obtained by using the experiments terminated at 24 h.
- Approach 2: We make use of the ABC posterior of {*D*_(0–24)_, *q*_(0–24)_} previously obtained from the experiments terminated at 24 h, and only infer the values of {*D*_(24–48)_, *q*_(24–48)_} by matching on the summary statistics at 48 h. To achieve this, for each initial cell density, we fit a bivariate normal distribution to the ABC joint posterior distributions of {*D*_(0–24)_, *q*_(0–24)_}. To perform a model simulation, we draw a parameter set from the bivariate normal distribution for the first stage, and another parameter set from the uniform prior for {*D*_(24–48)_, *q*_(24–48)_} for the second stage.

We use the same uniform prior for {*D*_(24–48)_, *q*_(24–48)_} in the two approaches. The second approach has the advantage that the SMC-ABC algorithm only needs to search over the parameter space of 24–48 h, {*D*_(24–48)_, *q*_(24–48)_}. Thus, we expect the second approach to be faster and more efficient. For each joint posterior distribution of {*D*_(0–24)_, *q*_(0–24)_}, we assess the bivariate normality assumption using a Q-Q plot of chi-square quantiles against the squared Mahalanobis distance [43]. The Q-Q plots suggest that the bivariate normality assumption is reasonable for both initial cell densities.

We found that the ABC posterior distributions of {*D*_(24–48)_, *q*_(24–48)_} in the two approaches are indistinguishable. However, the second approach is more efficient in terms of computational time. Therefore, for all experimental conditions in the two scenarios, we first obtain estimates for periods 0–24 h and 0–48 h then use the second approach to obtain estimates for 24–48 h.

**Fig 6:**
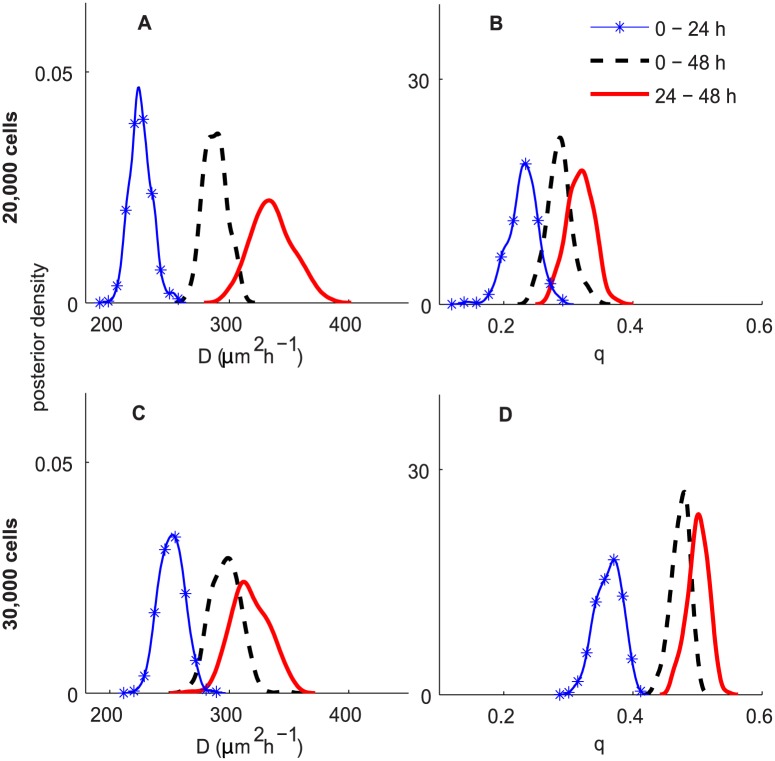
ABC posterior distributions for *D* and *q* for experiments in Scenario 1. Subfigures A–B and C–D correspond to the experiments initialised with 20,000 and 30,000 cells, respectively. In all subfigures, the blue curve with markers, the black dashed and the red solid curves correspond to the ABC posteriors for 0–24 h, 24–48 h and 0–48 h, respectively.

A comparison of *D* and *q* for different time periods is shown in Fig 6. Results in Fig 6A–6D correspond to experiments initiated with 20,000 and 30,000 cells, respectively. We observe that the estimated posterior distributions of *D*_(0–24)_ and *D*_(24–48)_ are non-overlapping, which implies that estimates of cell diffusivity are significantly different for the two periods of the experiment.

Comparing the posterior estimates of *D* for different *C*(0) suggests that values of *D* for the 30,000 initial cell density experiment is higher than for those in the 20,000 initial cell density experiment during the period 0–24 h. However, the difference is insignificant for the period 24–48 h and for the entire period 0–48 h. These findings indicate that estimates of cell diffusivity depend less on the initial cell density for longer experiments.

In contrast, the posterior estimates of cell-to-cell adhesion strength, *q*, for different *C*(0) are substantially different for all three periods. In particular, the estimates of *q* for the experiments initiated with 30,000 cells are higher than the corresponding values from the experiments initiated with 20,000 cells. This implies that estimates of cell-to-cell adhesiveness depend on initial cell densities. The higher the initial density, the stronger the cell-to-cell adhesion strength. In the literature, several studies have investigated the role of cell-to-cell adhesion in collective cell spreading [44–46] by matching the cell density profiles between the experimental data and the model simulation with several values of *q*. The previous approach is limited in that it can only give a point estimate of *q* and provide no insight into the uncertainty in the estimate or the correlation between *D* and *q*. Therefore, this study is the first attempt to provide a systematic approach to jointly infer the values of *D* and *q*, and compare the distributions of *D* and *q* for different experimental conditions.

### Scenario 2: Experiments without Mitomycin-C pre-treatment

To analyse the second set of experiments, we consider two approaches: (i) assuming that the values of *D* and *q* in the two experimental scenarios are completely unrelated, and thus, inferences of *D*, *q* and *λ* are based solely on the experimental data in Scenario 2, and (ii) assuming that the values of *D* and *q* from Scenario 1 are equal to those of *D* and *q* in Scenario 2. For the latter approach, we adopt a Bayesian sequential learning approach and use the posterior distribution of *D* and *q* from Scenario 1 as the prior for *D* and *q* for the corresponding experiments in Scenario 2.

#### Uninformative priors for *D*, *q* and *λ*

We aim to obtain an approximate joint posterior distribution for our model parameters when little prior biological knowledge about them is assumed. For all cases, we observe that all the pilot summary statistics are informative for *D*, however, for *q* and *λ*, the largest two radii were not significant in the regression.

**Fig 7:**
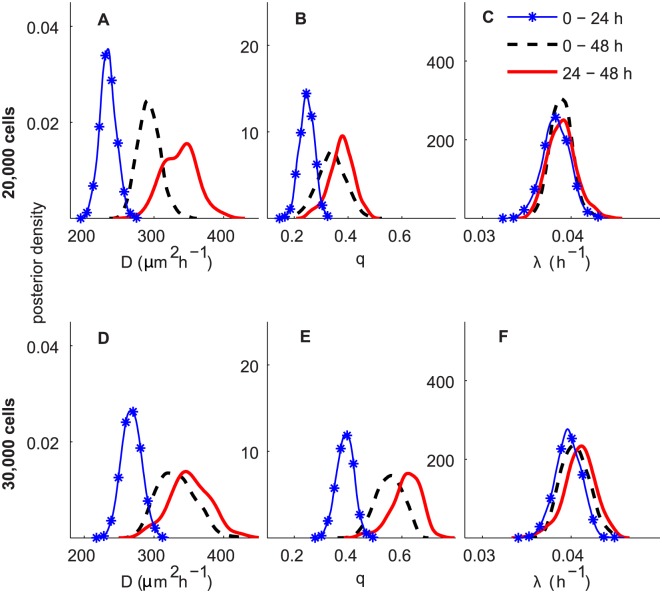
ABC posterior distributions for *D*, *q* and *λ* for experiments in Scenario 2, using uninformative priors for all parameters. Subfigures A–C and D–F correspond to the ABC posterior estimates for the experiments initiated with 20,000 and 30,000 cells, respectively. In all subfigures, the blue curve with markers, the black dashed and the red solid curves represent the ABC posterior distribution for 0–24 h, 0–48 h and 24–48 h, respectively.

A summary of the ABC posterior distributions of *D*, *q* and *λ* resulting from the ABC analyses with the derived summary statistics and uniform priors, *P*_*m*_ ∼ *U*(0,1), *q* ∼ *U*(0,1) and *P*_*p*_ ∼ *U*(0,1), corresponding to *D* ∼ *U*(0,2025) µm^2^h^−1^ and *λ* ∼ *U*(0,25)h^−1^, are given in Table 2 and a comparison of *D*, *q* and *λ* for the three time periods (0–24 h, 24–48 h and 0–48 h) are presented in Fig 7. Results in Fig 7A–7F correspond to the experiments initiated with 20,000 and 30,000 cells, respectively. The MCSE, for all cases, for *E*[*D*], *E*[*q*] and *E*[*λ*] are relatively small, less than 0.3%, 0.5% and 0.2% of the estimate of its expected values, respectively.

**Table 2:** ABC posterior summary for *D*, *q* and *λ* for all experiments in Scenario 2, using uninformative priors for *D*, *q* and *λ*. Results shown include the posterior mean (and the 90% CI in the parentheses) and the coefficient of variation, CV.

*C*(0)	*T*(*h*)	*E*[*D*] (90% CI) (µm^2^h^−1^)	CV(*D*)(%)	*E*[*q*]	CV(*q*)(%)	*E*[*λ*] (90% CI) ×10^−2^ (h^−1^)	CV(*λ*)(%)
20,000	0—24	234.0 (215.4, 253.9)	4.9	0.25 (0.21, 0.29)	10.7	3.83 (3.58, 4.08)	4.0
	0—48	292.9 (267.8, 321.2)	5.5	0.34 (0.25, 0.42)	15.3	3.89 (3.69, 4.09)	3.2
	24—48	336.9 (299.7, 377.1)	7.2	0.38 (0.29, 0.44)	11.8	3.90 (3.66, 4.18)	3.9
30,000	0—24	267.8 (245.1, 291.2)	5.2	0.39 (0.34, 0.44)	7.9	3.97 (3.72, 4.20)	3.6
	0—48	332.0 (293.1, 373.8)	7.9	0.51 (0.47, 0.63)	12.4	4.04 (3.80, 4.30)	3.8
	24—48	351.2 (296.8, 395.1)	8.1	0.61 (0.52, 0.69)	8.8	4.06 (3.77, 4.38)	4.2

The results in Table 2 indicate that we are able to obtain reasonably precise estimates for all *D*, *q* and *λ* based on the information incorporated initially through our pilot summary statistics. The CV for *λ* is fairly small, less than 5%, for all cases. This proposed method, therefore, overcomes the limitation in the previous work [16], which demonstrated that *λ* cannot be identified when precise prior information about the parameters is unavailable. The reason is that, here, for each experiment, we include information about the percentages of isolated cells and the cell counts for six sub-regions, whereas the previous analyses [16] solely used the leading edge. This also provides another strategy to obtain precise estimates of *λ* besides the technique proposed in [16] by incorporating information from experiments with and without Mitomycin C pre-treatment. The latter approach assumes that the common parameters are the same for different experimental scenarios, which may not be appropriate for all types of cells. The CV for *D* and *q* for all experimental conditions are also small, between 5—10% and 8—16%, respectively.

We observe a similar trend of time-dependence for *D* and *q* as in Fig 6 for Scenario 1. In summary, in both experimental scenarios, we observe a consistent time-dependence in our estimate of *D* and *q* suggesting that cell motility is slower in the first day duration relative to the second day. Interestingly, the values of *λ* remain similar over time, for both initial cell densities. The estimates of *E*[*λ*] are in the range 3.83 − 4.04 × 10^−2^ h^−1^. This gives an expected doubling time for melanoma cells of 17–18 h. It is also noted that our estimates of *λ* for the 20,000 initial cell experiments are slightly higher than the previously reported estimates [23], although the estimates of *λ* for the experiments initiated with 30,000 cells are similar.

We also found a similar trend of density-dependence for estimates of *D* and *q* as observed in Scenario 1. Comparing the posterior estimates of *λ* for different values of initial cells suggests that the estimates of *λ* for the 30,000 initial cell experiments are slightly higher than the 20,000 initial cell experiments. However, the differences are quite small.

#### Informative priors for *D*, *q* and an uninformative prior for *λ*

Although we are able to obtain reasonably precise estimates of *D*, *q* and *λ* using uninformative priors on all parameters, we wish to investigate whether additional information about model parameters is obtained when we combine information from the two experimental scenarios. The Bayesian sequential learning approach allows us to incorporate information from previous experiments in a principled way. This is similar to the approach used in [16]. Assuming that the values of *D* and *q* are the same in the two experimental scenarios, we use the posterior distributions of *D* and *q* in the first experimental scenario as informative priors for *D* and *q* in the second experimental scenario. To achieve this, we fit a bivariate normal distribution to each joint posterior distribution of *D* and *q* reported in Scenario 1, and use this bivariate normal distribution as an informative prior for *D* and *q* for the corresponding experiment in Scenario 2. For each joint posterior distribution of *D* and *q*, we assess the bivariate normality assumption using a Q-Q plot of chi-square quantiles against the squared Mahalanobis distance [43]. The Q-Q plots indicate that the bivariate normality assumption is reasonable for all cases (results not shown).

A summary of the ABC posteriors for *D*, *q* and *λ* for all experimental combinations is presented in Table 3. The MCSE, for all cases, for *E*[*D*], *E*[*q*] and *E*[*λ*] are relatively small, less than 0.3% of the mean value estimates. Our results show that, for all cases, the CV for *D* and *λ* using the bivariate normal prior is smaller than the corresponding CV reported using the previous approach (uninformative priors for all parameters). This implies that we obtain more precision for all *D* and *λ* by incorporating information from the two experimental scenarios.

**Table 3:** ABC posterior summary for *D*, *q* and *λ* for all experiments in Scenario 2, using informative priors for *D*, *q* and an uninformative prior for *λ*. Results shown include the posterior mean (and the 90% CI in the parentheses) and the coefficient of variation, CV.

*C*(0)	*T*(*h*)	*E*[*D*] (90% CI)µm^2^h^−1^	CV(*D*)(%)	*E*[*q*] (90% CI)	CV(*q*)(%)	*E*[*λ*] (90% CI) ×10^−2^h^−1^	CV(*λ*)(%)
20,000	0—24	231.9 (220.6, 243.0)	3.0	0.24 (0.21, 0.27)	7.5	3.83 (3.62, 4.04)	3.2
	0—48	286.5 (274.3, 301.1)	2.8	0.29 (0.26, 0.34)	8.0	3.81 (3.71, 3.93)	1.8
	24—48	334.9 (311.6, 356.7)	4.1	0.33 (0.29, 0.37)	6.7	3.90 (3.69, 4.10)	3.2
30,000	0—24	260.2 (246.3, 274.9)	3.4	0.37 (0.34, 0.41)	5.2	3.94 (3.75, 4.13)	2.9
	0—48	300.9 (284.9, 316.2)	3.2	0.48 (0.46, 0.51)	3.7	3.95 (3.81, 4.09)	2.1
	24—48	329.2 (307.6, 352.0)	4.3	0.50 (0.47, 0.53)	4.0	3.90 (3.66, 4.10)	3.5

Comparing the posterior distributions of *D* obtained using the previous approach (uninformative priors for all parameters) and those results obtained by specifying an uninformative prior for *λ* and an informative bivariate normal prior for *D* and *q* show that we infer *D* reasonably well for both sets of priors. The estimates of *E*[*D*] are very similar regardless of these choices of priors (Table 3); however, the CV for *D* is smallest when we combine the information from the two experiments. A similar trend is also observed for the experiments initiated with 30,000 cells.

**Fig 8:**
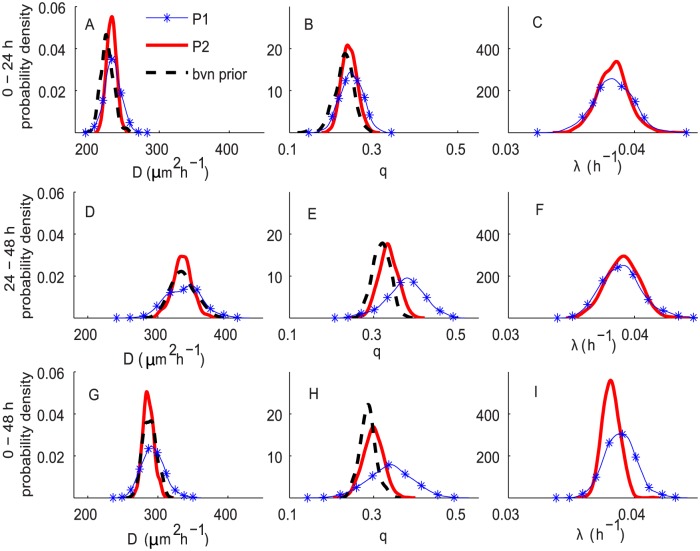
ABC posterior distributions for *D*, *q* and *λ* for experiments in Scenario 2. These results correspond to *C*(0) = 20,000. Subfigures A–C, D–F and G–I correspond to the ABC posterior estimates for the experiments at 0–24 h, 24–48 h and 0–48 h, respectively. In all subfigures, the blue curves with markers, P1, correspond to the approach using uninformative priors for all parameters. The red solid curves, P2, correspond to the approach using informative priors for *D*, *q* and an uninformative prior for *λ*. The fitted bivariate normal priors, bvn prior, are shown as black dashed curves.

Regarding the cell-to-cell adhesion, we observe additional information about *q* only for the period of 0–24 h. For the periods of 24–48 h and 0–48 h, there is an indication that the posterior estimates of *q* from the two experiments are slightly different. The posteriors of *q* resulting from the bivariate normal prior for *D* and *q* are shifted toward the posteriors obtained by using uninformative priors (Fig 8B, 8E and 8H). This could be explained by noting that the densities of the two experiments are potentially different after 24 h, since the second experiment involves cell proliferation. Thus, the posterior estimates of cell-to-cell adhesion strength in the second experiments are suggested to be slightly higher than those in the first experiment.

The ABC posteriors for *λ* (Fig 8C, 8F and 8I) obtained from the two approaches are similar, except that the posteriors obtained by placing an informative bivariate normal prior on *D* and *q* are narrower. In summary, these results suggest that, for melanoma cells, if we are given some information about *D* and *q*, we can gain more precision in all estimations of *D* and *λ*. However, without some prior information about *D* and *q*, the proposed ABC approach and the summarisation of the experimental images used can produce reasonably precise estimates for *D*, *q* and *λ* from a single assay. A similar trend is also observed for the 30,000 cell experiments (S1 Fig).

**Fig 9:**
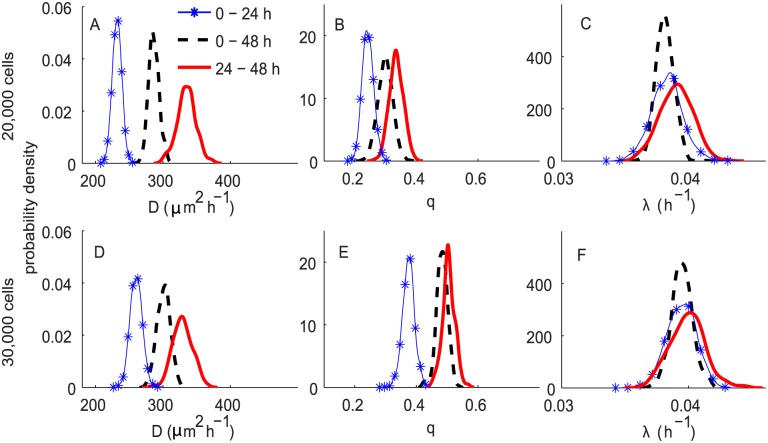
ABC posterior distributions for *D*, *q* and *λ* for experiments in Scenario 2, using informative priors for *D*, *q* and an uninformative prior for *λ*. Subfigures A–C and D–F correspond to the ABC posterior estimates for the experiments initiated with 20,000 and 30,000 cells, respectively. In all subfigures, the blue curve with markers, the black dashed and the red solid curves represent the ABC posterior distribution for 0–24 h, 0–48 h and 24–48 h, respectively.

Fig 9 shows a comparison of ABC posterior distributions of *D*, *q* and *λ* with respect to different time periods. We observe a similar trend of time-dependency and density-dependency to that observed in Figs 6 and 7. For both initial cell densities, *D* and *q* appear to depend on the time period, whereas the values of *λ* remain almost constant over time.

## Discussion

Quantifying the underlying mechanisms that drive the expansion of melanoma cell colonies such as migration, proliferation, and cell-to-cell adhesion is important for developing a systematic approach to assessing the effectiveness of a potential treatment. Typical approaches to parameter estimation often use a deterministic framework [4–7, 23] and only produce point estimates. There is, therefore, a risk that future model projections based on such point estimates could be made with undue confidence.

In this paper, we present a new ABC algorithm to estimate *D*, *q* and *λ* which represent the cell motility, the cell-to-cell adhesion strength and the cell proliferation rate, respectively. To the best of our knowledge, this is the first time that joint inferences have been obtained for all three parameters in a discrete stochastic model describing expanding melanoma cell colonies, using data from a single assay. The new ABC algorithm shows favourable performance relative to state-of-the-art algorithms and together with our derived summary statistics, we can estimate all model parameters precisely across different scenarios, even when a vague prior is used (Tables 1 and 2). This emulates a situation in which virtually no biological knowledge about *D*, *q* and *λ* is assumed. Furthermore, the methodology developed here overcomes the limitation in the previous work [16], which demonstrated that without prior information about *D*, *λ* cannot be identified using solely leading edge data.

The methodology proposed here allows us to obtain inferences for *D*, *q* and *λ* in a fully Bayesian framework. The resulting posterior distributions enable us to quantify the associated uncertainty with the parameter estimates which can not be achieved using a deterministic approach. Furthermore, comparing the distributions of *D*, *q* and *λ* (Figs 6, 7 and 9) provides insight into the dependency of the parameter posterior estimates on the experimental elapsed time and on the initial number of cells. Thus, our work adds significant extra information about the parameters relative to the previous analyses [23]. Another advantage of using an ABC approach is the possibility of combining information from the two experiments in a principled way. This approach is shown to be useful in our previous work [16]. Here, it also enables us to gain additional information for *D* and *λ*.

We acknowledge that our discrete individual-based model, which is straightforward to implement and computationally cheap, makes an assumption that cell diffusivity is constant. Although the density dependence is less pronounced for experiments terminated at 48 h, it suggests that the underlying assumption of a constant diffusion coefficient *D* is violated. Thus, it is suggested that the use of a non-linear diffusion coefficient, where *D* is a function of cell density, *D*(*C*), may be more appropriate. In particular, using non-linear diffusion coefficients is shown to provide a better description of the collective behaviour of a cell population in a lattice-free model [47] and a model with complex contact interactions [48]. We expect that implementation of the ABC approach for these models will lead to further research.

It should also be noted that [23] obtained point estimates of *D*, *q* and *λ* separately; *D* and *q* from the experiments with cell proliferation suppressed, and *λ* from experiments with cell proliferation. Thus, this approach may not be applicable if one does not have access to this kind of detailed experimental data sets. Furthermore, results from our analyses also indicate that cell-to-cell adhesion may differ between the two scenarios. In particular, the values of cell-to-cell adhesion is slightly higher for the experiments with cell proliferation occurring, due to the increasing cell population. Thus, we suggest that future studies should consider estimating all parameters simultaneously.

One particular finding from our analysis is that the posterior distributions of *D* and *q* consistently depend on the experimental time period, whereas the posterior distribution of *λ* is approximately time constant. This finding is in agreement with the results of [16] for 3T3 fibroblast cells, however, this feature has not been investigated elsewhere. As demonstrated earlier, this effect is significant and should be included when modelling mechanisms governing the expansion of cell colonies in future research. To achieve this, we suggest that experimental data should be collected at several time points and to optimally do this we leave for future research.

In addition, our ABC algorithm together with the derived summary statistics could also be implemented in a model selection algorithm to distinguish between discrete lattice-based and lattice-free models describing the expansion of cell colonies. In lattice-free models, agents are allowed to migrate and proliferate in a continuous domain, and the direction of movement is a continuous variable [10]. Thus this model is considered to be more realistic than the lattice-based model.

## Supporting Information

S1 Text (PDF)

S1 Fig (EPS)

## References

[1] American Cancer Society, Cancer Facts & Figures (2011). Available: http://www.cancer.org/Research/CancerFactsFigures/cancer-facts-figures-2011 Retrieved: June 29, 2015.

[2] Australian Institute of Health and Welfare and Australasian Associate of Cancer Registries (2012). Cancer in Australia: an overview. Cancer series no. 74. Cat. no. CAN 70. Canberra: AIHW.

[3] Decaestecker C., Debeir O., Van Ham P. and Kiss R. (2007). Can anti-migratory drugs be screened in vitro? A review of 2D and 3D assays for the quantitative analysis of cell migration. Medicinal Research Reviews, 27(2):149–176. 16888756 10.1002/med.20078

[4] Pettet G. J., Byrne H. M., McElwain D. L. S. and Norburry J. (1996). A model of wound-healing angiogenesis in soft tissue. Mathematical Biosciences, 136(1):35–63. 10.1016/0025-5564(96)00044-2 8755336

[5] Savla U., Olson L. E. and Waters C. M. (2004). Mathematical modeling of airway epithelial wound closure. Journal of Applied Physiology, 96(2):566–574. 10.1152/japplphysiol.00510.2003 14715680

[6] Maini P. K., McElwain D. L. S. and Leavesley D. I. (2004). Traveling wave model to interpret a wound-healing cell migration assay for human peritoneal mesothelial cells. Tissue Engineering, 10(3-4):475–482. 10.1089/107632704323061834 15165464

[7] Swanson K. R. (2008). Quantifying glioma cell growth and invasion in vitro. Mathematical and Computer Modelling, 47(5):638–648. 10.1016/j.mcm.2007.02.024

[8] Callaghan T., Khain E., Sander L. M. and Ziff R. M. (2006). A stochastic model for wound healing. Journal of Statistical Physics, 122(5):909–924. 10.1007/s10955-006-9022-1

[9] Khain E., Sander L. M. and Schneider-Mizell C. M. (2007). The role of cell-cell adhesion in wound healing. Journal of Statistical Physics, 128(1):209–218. 10.1007/s10955-006-9194-8

[10] Plank M. J. and Simpson M. J. (2012). Models of collective cell behaviour with crowding effects: comparing lattice-based and lattice-free approaches. Journal of the Royal Society Interface, 9(76):2983–2996. 10.1098/rsif.2012.0319 PMC3479911 22696488

[11] Murray P. J., Walter A., Fletcher A. G., Edwards C. M., Tindall M. J. and Maini P. K. (2011). Comparing a discrete and continuum model of the intestinal crypt. Physical Biology, 8(2):026011 10.1088/1478-3975/8/2/026011 21411869 PMC3164594

[12] Beaumont M. A., Zhang W., and Balding D. J. (2002). Approximate Bayesian computation in population genetics. Genetics, 162(4):2025–2035. 12524368 10.1093/genetics/162.4.2025 PMC1462356

[13] Drovandi C. C. and Pettitt A. N. (2011). Using approximate Bayesian computation to estimate transmission rates of nosocomial pathogens. Statistical Communications in Infectious Diseases, 3(1):2 10.2202/1948-4690.1025

[14] Tanaka M. M. and Francis A. R. and Luciani F. and Sisson S. A. (2006). Using approximate Bayesian computation to estimate tuberculosis transmission parameters from genotype data. Genetics, 173(3):1511–1520. 10.1534/genetics.106.055574 16624908 PMC1526704

[15] Cameron E. and Pettitt A. (2012). Approximate Bayesian computation for astronomical model analysis: A case study in galaxy demographics and morphological transformation at high Redshift. Monthly notices of the Royal Astronomical Society, 425(1):44–65. 10.1111/j.1365-2966.2012.21371.x

[16] Vo B. N., Drovandi C. C, Pettitt A. N. and Simpson M. J. (2015). Quantifying uncertainty in parameter estimates for stochastic models of collective cell spreading using approximate Bayesian computation. Mathematical Biosciences, 263:133–142. 10.1016/j.mbs.2015.02.010 25747415

[17] Drovandi C. C. and Pettitt A. N. (2011). Estimation of parameters for macroparasite population evolution using approximate Bayesian computation. Biometrics, 67(1):225–233. 10.1111/j.1541-0420.2010.01410.x 20345496

[18] Beaumont M. A., Cornuet J., Marin J. and Robert C. P. (2009). Adaptive approximate Bayesian computation. Biometrika, 96(4):983–990. 10.1093/biomet/asp052

[19] Toni T., Welch D., Strelkowa N., Ipsen A. and Stumpf M. P. H. (2009). Approximate Bayesian computation scheme for parameter inference and model selection in dynamical systems. Journal of the Royal Society Interface, 6(31):187–202. 10.1098/rsif.2008.0172 PMC2658655 19205079

[20] Lenormand M., Jabot F. and Deffuant G. (2013). Adaptive approximate Bayesian computation for complex models. Computational Statistics, 28(6):2777–2796. 10.1007/s00180-013-0428-3

[21] Blum M. G. B. (2010). Approximate Bayesian Computation: a non-parametric perspective. Journal of the American Statistical Association, 105(491):1178–1187. 10.1198/jasa.2010.tm09448

[22] Fearnhead P. and Prangle D. (2012). Constructing summary statistics for approximate Bayesian computation: Semi-automatic ABC (with discussion). Journal of the Royal Statistical Society: Series B (Statistical Methodology), 74(3):419–474.

[23] Treloar K. K., Simpson M. J., Haridas P., Manton K. J., Leavesley D. I., McElwain D. L. S. and Baker R. E. (2013). Multiple types of data are required to identify the mechanisms influencing the spatial expansion of melanoma cell colonies. BMC Systems Biology, 7:137 10.1186/1752-0509-7-137 24330479 PMC3878834

[24] Debeir O., Mégalizzi V., Warzée N., Kiss R. and Decaestecker C. (2008). Videomicroscopic extraction of specific information on cell proliferation and migration in vitro. Experimental Cell Research, 314(16):2985–2998. 10.1016/j.yexcr.2008.06.010 18598694

[25] Pope J. H., Morrison L., Moss D. J., Parsons P. G. and Mary S. R. (1979). Human malignant melanoma cell lines. Pathology, 11(2):191–195. 10.3109/00313027909061945 460945

[26] Whitehead R. and Little J. H. (1973). Tissue culture studies on human malignant melanoma. Pigment Cell, 1:382–389.

[27] Sadeghi M., Seitz B., Hayashi S. LaBree L. and McDonnell P. (1998). In vitro effects of Mitomycin-C on human keratocytes. Journal of Refractive Surgery, 14:534–540. 9791820 10.3928/1081-597X-19980901-11

[28] MathWorks, Image processing toolbox. (2012). Retrieved: January 5, 2015.

[29] Simpson M. J., Landman K. A., and Hughes B. D. (2010). Cell invasion with proliferation mechanisms motivated by time-lapse data. Physica A: Statistical Mechanics and its Applications, 389(18):3779–3790. 10.1016/j.physa.2010.05.020

[30] Chowdhury D., Schadschneider A. and Nishinari K. (2005). Physics of transport and traffic phenomena in biology: from molecular motors and cells to organisms. Physics of Life Reviews, 2(4):318–352. 10.1016/j.plrev.2005.09.001

[31] Wilkinson D. J. (2011). Stochastic Modelling for Systems Biology. CRC Press.

[32] Gillespie D. T. (1997). Exact stochastic simulation of coupled chemical reactions. Journal of Physical Chemistry, 81(25):2340–2361. 10.1021/j100540a008

[33] Marjoram P., Molitor J., Plagnol V., and Tavaré S. (2003). Markov chain Monte Carlo without likelihoods. Proceedings of the National Academy of Sciences of the United States of America, 100(26):15324–15328. 10.1073/pnas.0306899100 14663152 PMC307566

[34] Bortot P., Coles S. G. and Sisson S. A. (2007). Inference for stereological extremes. Journal of the American Statistical Association, 102(477):84–92. 10.1198/016214506000000988

[35] Sisson S. A. and Fan Y. (2011). MCMC handbook, chapter Likelihood-free Markov chain Monte Carlo, pages 313–335. Chapman & Hall.

[36] Sisson S. A., Fan Y., and Tanaka M. M. (2007). Sequential Monte Carlo without likelihoods. Proceedings of the National Academy of Sciences of the United States of America, 104(6):1760–1765. 10.1073/pnas.0607208104 17264216 PMC1794282

[37] Csilléry K., Blum M. G. B., Gaggiotti O. E. and François O. (2010). Approximate Bayesian computation (ABC) in practice. Trends in Ecology & Evolution, 25(7):410–418. 10.1016/j.tree.2010.04.001 20488578

[38] Sisson S. A., Fan Y. and Tanaka M. M. (2009). Correction for Sisson et al., Sequential Monte Carlo without likelihoods. Proceedings of the National Academy of Sciences of the United States of America, 106(39):16889–16890. 10.1073/pnas.0908847106 PMC1794282 17264216

[39] Del Moral P., Doucet A. and Jasra A. (2012). An adaptive sequential Monte Carlo method for approximate Bayesian computation. Statistics and Computing, 22(5):1009–1020. 10.1007/s11222-011-9271-y

[40] Blum M. G. B., Nunes M. A., Prangle D. and Sisson S. A. (2013). A comparative review of dimension reduction methods in approximate Bayesian computation. Statistical Science, 28(2):189–208. 10.1214/12-STS406

[41] Geyer C. J. (1992). Practical Markov chain Monte Carlo. Statistical Science, 7(4):473–483. 10.1214/ss/1177011147

[42] Doucet A. and Godsill S. and Andrieu C. (2000). On sequential Monte Carlo sampling methods for Bayesian filtering. Statistics and Computing, 10(3):197–208. 10.1023/A:1008935410038

[43] Korkmaz S., Goksuluk D. and Zararsiz G. (2014). MVN: an R package for assessing multivariate normality. R Journal, 6(2):151–162.

[44] Deroulers C., Aubert M., Badoual M. and Grammaticos B. (2009). Modeling tumor cell migration: from microscopic to macroscopic models. Physical Review E, 79(3):031917 10.1103/PhysRevE.79.031917 19391981

[45] Anderson A. R. A. (2005). A hybrid mathematical model of solid tumour invasion: The importance of cell adhesion. Mathematical Medicine and Biology, 22(2):163–186. 10.1093/imammb/dqi005 15781426

[46] Simpson M. J., Towne C., McElwain D. L. S. and Upton Z. (2010). Migration of breast cancer cells: Understanding the roles of volume exclusion and cell-to-cell adhesion. Physical Review E, 82:041901 10.1103/PhysRevE.82.041901 21230307

[47] Dyson L., Maini P. K. and Baker R. E. (2012). Macroscopic limits of individual-based models for motile cell populations with volume exclusion. Physical Review E, 86:031903 10.1103/PhysRevE.86.031903 23030940

[48] Fernando A. E., Landman K. A and Simpson M. J. (2010). Nonlinear diffusion and exclusion processes with contact interactions. Physical Review E, 81:011903 10.1103/PhysRevE.81.011903 20365395

