# Supplementary material for: Melanoma Cell Colony Expansion Parameters Revealed by Approximate Bayesian Computation
Source: PLoS Comput Biol. 2015 Dec 7;11(12):e1004635. doi: 10.1371/journal.pcbi.1004635 (PMC4671693; doi:10.1371/journal.pcbi.1004635)
Supplement: S1 Text — (PDF) [file pcbi.1004635.s001.pdf]

S1 Text: Supporting Information

Radius of expanding cell population

Summary of Treloar et al. [1] experimental data for all population-scale images in two different experimental scenarios: (1) Mitomycin-C is applied as a treatment to suppress cell proliferation, and (2) no treatment is applied. Measurements of the radius of the entire expanding cell populations for all experimental conditions are given below.

Table S1: Radius of expanding cell populations. Here $C(0)$ and T are the initial cell density and the experimental termination time (in hours), respectively.

$C(0)$	T (h)	Radius (mm) with Mitomycin-C pre-treatment			Radius (mm) without Mitomycin-C pre-treatment		
20,000	0	3.1801	3.1845	3.1847	3.1821	3.1799	3.1850
	24	3.2619	3.2535	3.2713	3.3036	3.3105	3.3165
	48	3.3518	3.3590	3.3605	3.4557	3.4583	3.4590
30,000	0	3.1840	3.1826	3.1810	3.1910	3.1800	3.1830
	24	3.3176	3.3020	3.3062	3.3322	3.3393	3.3360
	48	3.3574	3.3629	3.3679	3.5652	3.5410	3.5760

Cell count

The total number of cells in six sub-regions (averaged over three replicates) for all experimental conditions. Here $C(0)$ and T are the initial cell density and the experimental termination time (in hours), respectively.

Table S2: Cell counts. Number of cells in six sub-regions in a transect image for two experimental scenarios, averaged over three replicates.

$C(0)$	T (h)	Number of cells with Mitomycin-C pre-treatment						Number of cells without Mitomycin-C pre-treatment					
20,000	0	231	241	252	237	217	236	231	229	239	243	253	231
	24	242	227	236	245	237	228	444	445	407	457	440	424
	48	245	248	236	241	233	227	653	631	647	676	644	654
30,000	0	344	330	345	353	350	340	354	349	361	335	344	331
	24	359	337	340	358	346	359	597	572	587	566	564	595
	48	364	363	381	374	371	366	791	809	795	815	800	768

Percentages of isolated cells

The percentages of isolated cells in six sub-regions (averaged over three replicates). Here $C(0)$ and T are the initial cell density and the experimental termination time (in hours), respectively.

Table S3: Percentages of isolated cells in six sub-regions in a transect image for two experimental scenarios, averaged over three replicates.

$C(0)$	T (h)	Percentages of isolated cells (%)											
		with Mitomycin-C pre-treatment						without Mitomycin-C pre-treatment					
20,000	0	34.7	42.2	39.4	47.13	34.49	41.6	41.04	42.8	39.2	36.4	34.6	37.1
	24	29.6	37.4	36.9	36.1	35.7	32.8	12.3	11.8	12.6	11.2	11.0	10.1
	48	29.8	32.6	33.2	31.9	30.6	30.5	2.4	2.6	2.3	2.0	3.1	2.4
30,000	0	24.2	25.8	26.8	26.1	25.1	23.0	23.1	23.4	25.9	23.7	20.2	25.4
	24	16.5	15.4	17.8	16.0	17.5	18.6	3.2	4.1	4.2	4.4	4.8	3.2
	48	10.0	13.3	11.2	8.8	11.6	12.61	0.6	0.6	0.8	0.6	0.5	0.9

Simulation algorithm for the discrete stochastic model

The simulation algorithm for the discrete stochastic model describing the expansion of cell colonies is shown in Algorithm S1, with the function $\lfloor\cdot\rfloor$ denotes the integer part of a real number.

1 Initialise the simulated experiment at $t = 0$, set $\tau = 0.04$ and set values for $C(0)$, $N_{steps},\ P_m,\ P_p,\ q$
2 **for** $i = 1$ **to** N_{steps} **do**
3 | Let $C(t)$ be the total number of agents at time t
4 | Let $N_{move} = \lfloor(C(t) \times P_m)\rfloor$, $R = C(t) \times P_m - N_{move}$
5 | **for** $j = 1$ **to** N_{move} **do**
6 | | Draw $r \sim$ discrete-uniform $(1, C(t))$ to choose which agent to move
7 | | Compute the number of occupied neighbours, a
8 | | **if** uniform (0,1) $\leq (1-q)^a$ **then**
9 | | | Draw $d \sim$ uniform (0, 1) to choose the target site
10 | | | Move the agent if the target is vacant
11 | | **end**
12 | **end**
13 | Choose an agent to undertake a motility event with probability $R \times (1-q)^a$ (i.e. repeat steps 7–12 above)
14 | Let $N_p = \lfloor(C(t) \times P_p)\rfloor$, $R = C(t) \times P_p - N_p$
15 | **for** $j = 1$ **to** N_p **do**
16 | | Draw $r \sim$ discrete-uniform $(1, C(t))$ to choose which agent to proliferate
17 | | Draw $d \sim$ uniform (0, 1) to choose the target site for the new daughter agent
18 | | Deposit the daughter agent only if the target is vacant and update $C(t)$
19 | **end**
20 | Choose an agent to undertake a proliferation event with probability R
21 | Update population of agents to time $t = t + \tau$
22 **end**

Algorithm S1: Simulation algorithm for the discrete stochastic model that includes mechanisms for cell motility, cell proliferation and cell-to-cell adhesion. If cell proliferation is suppressed then lines 14–20 can be omitted.

ASMC Algorithm

In our ASMC algorithm, $N_\alpha = \lfloor \alpha N \rfloor$ is the number of particles to keep at each iteration among the N particles, where $\alpha \in [0, 1]$ and the stopping criterion is either the minimal acceptance rate, $p_{acc_{min}}$, or a target tolerance, ϵ_{final}. A reasonable choice for α is 0.5 [2]. Here $\mathcal{N}(\cdot, \cdot)$ denotes the multivariate normal distribution.

Given N, N_α, $p_{acc_{min}}$, ϵ_{final}.
Set $p_{acc} = 1, \quad k = 0$
for $i = 1$ **to** N **do**
    Simulate $\theta_i^{(k)} \sim \pi(\theta)$ and $y_{sim} \sim f(\cdot|\theta_i^{(k)})$
    $\rho_i^{(k)} = \rho(S(y_{obs}), S(y_{sim}))$
    $w_i^{(k)} = \frac{1}{N}$
end
$\epsilon^{(k)} = \max_{i=1,\ldots,N} \{\rho_i^{(k)}\}$
while *($p_{acc} > p_{acc_{min}}$) and ($\epsilon^{(k)} > \epsilon_{final}$)* **do**
    Sort the particle set $(\theta_i^{(k)}, \rho_i^{(k)})_{i=1}^N$ by $\rho_i^{(k)}$, such that $\rho_1^{(k)} \leq \rho_2^{(k)} \leq ... \leq \rho_N^{(k)}$
    Normalise the weights $W_i^{(k)} = w_i^{(k)} / \sum_{j=1}^{N_\alpha} w_j^{(k)}$ for $i = 1, \ldots, N_\alpha$
    Set Σ_k as twice the weighted empirical covariance using $(\theta_i^{(k)}, W_i^{(k)})_{i=1}^{N_\alpha}$
    Set $\epsilon^{(k)} = \rho_{N-N_\alpha}^{(k)}$ and the number of trials, $N_{trials} = 0$
    for $i = N_\alpha + 1$ **to** N **do**
        while $\rho_i^{(k)} > \epsilon^{(k)}$ **do**
            Draw $\theta_i^\star$ from $(\theta_j^{(k)}, W_j^{(k)})_{j=1}^{N_\alpha}$
            Generate $\theta_i^{(k)} | \theta_i^\star \sim \mathcal{N}(\theta_i^\star,\ \Sigma_k)$ and simulate $y_{sim} \sim f(\cdot|\theta_i^{(k)})$
            Set $N_{trials} = N_{trials} + 1$
            Compute $\rho_i^{(k)} = \rho(S(y_{obs}), S(y_{sim}))$
        end
        Set $w_i^{(k)} = \frac{\pi(\theta_i^{(k)})}{\sum_{j=1}^{N_\alpha} W_j^{(k)} \mathcal{N}(\theta_i^{(k)}; \theta_j^{(k)}, \Sigma_k)}$
    end
    Set $p_{acc} = \frac{N - N_\alpha}{N_{trials}}$
    Normalise the weights $W_i^{(k)} = w_i^{(k)} / \sum_{j=N_\alpha+1}^{N} w_j^{(k)}$ for $i = N_\alpha + 1, \ldots, N$.
    Set $w_i^{(k+1)} = \frac{N_\alpha}{N} W_i^{(k)}$ for $i = 1, \ldots, N_\alpha$ and $w_i^{(k+1)} = \frac{N - N_\alpha}{N} W_i^{(k)}$ for $i = N_\alpha + 1, \ldots, N$
    Set $k = k + 1$
end

Algorithm S2: ASMC algorithm.

ABC posterior distributions for D, q and λ for experiments in Scenario 2, $C(0) = 30{,}000$ cells.

Fig. S1: ABC posterior distributions for D, q and λ for experiments in Scenario 2. These results correspond to $C(0) = 30{,}000$. Subfigures A–C, D–F and G–I correspond to the ABC posterior estimates for the experiments at 0–24 h, 24–48 h and 0–48 h, respectively. In all subfigures, the blue curves with markers, P1, correspond to the approach using uninformative priors for all parameters. The red solid curves, P2, correspond to the approach using informative priors for D, q and an uninformative prior for λ. The fitted bivariate normal priors, bvn prior, are shown as black dashed curves.

SI References

[1] Treloar, K. K., Simpson, M. J., Haridas, P., Manton, K. J., Leavesley, D. I., McElwain, D. L. S. and Baker, R. E. (2013). Multiple types of data are required to identify the mechanisms influencing the spatial expansion of melanoma cell colonies. BMC Systems Biology, 7:137.

[2] Drovandi, C. C. and Pettitt, A. N. (2011). Estimation of parameters for macroparasite population evolution using approximate Bayesian computation. Biometrics, 67(1):225–233.
